# Association Between a History of Nontyphoidal *Salmonella* and the Risk of Systemic Lupus Erythematosus: A Population-Based, Case-Control Study

**DOI:** 10.3389/fimmu.2021.725996

**Published:** 2021-11-23

**Authors:** Ting-Yu Tu, Chiu-Yu Yeh, Yao-Min Hung, Renin Chang, Hsin-Hua Chen, James Cheng-Chung Wei

**Affiliations:** ^1^ Department of Orthopedics, Kaohsiung Veterans General Hospital, Kaohsiung, Taiwan; ^2^ Department of Medicine, School of Medicine, Kaohsiung Medical University, Kaohsiung, Taiwan; ^3^ College of Health and Nursing, Meiho University, Pingtung, Taiwan; ^4^ Institute of Medicine, Chung Shan Medical University, Taichung, Taiwan; ^5^ Department of Internal Medicine, Kaohsiung Municipal United Hospital, Kaohsiung, Taiwan; ^6^ School of Medicine, National Yang Ming University, Taipei, Taiwan; ^7^ Department of Emergency Medicine, Kaohsiung Veterans General Hospital, Kaohsiung, Taiwan; ^8^ Division of Allergy, Immunology and Rheumatology, Division of General Internal Medicine, Department of Internal Medicine, Taichung Veterans General Hospital, Taichung, Taiwan; ^9^ Institute of Biomedical Science and Rong Hsing Research Centre for Translational Medicine, Chung Hsing University, Taichung, Taiwan; ^10^ Department of Industrial Engineering and Enterprise Information, Tunghai University, Taichung, Taiwan; ^11^ Institute of Public Health and Community Medicine Research Center, National Yang-Ming University, Taipei, Taiwan; ^12^ Graduate Institute of Integrated Medicine, China Medical University, Taichung, Taiwan; ^13^ Division of Allergy, and Institute of Medicine, Chung Shan, Medical University, Immunology and Rheumatology, Taichung, Taiwan

**Keywords:** nontyphoidal *Salmonella*, systemic lupus erythematosus, NHIRD, case-control study, immunology, NTS, SLE, epidemiology

## Abstract

**Objective:**

We investigated the correlation between nontyphoidal S*almonella* (NTS) infection and systemic lupus erythematosus (SLE) risk.

**Methods:**

This case-control study comprised 6,517 patients with newly diagnosed SLE between 2006 and 2013. Patients without SLE were randomly selected as the control group and were matched at a case-control ratio of 1:20 by age, sex, and index year. All study individuals were traced from the index date back to their NTS exposure, other relevant covariates, or to the beginning of year 2000. Conditional logistic regression analysis was used to analyze the risk of SLE with adjusted odds ratios (aORs) and 95% confidence intervals (CIs) between the NTS and control groups.

**Results:**

The mean age was 37.8 years in the case and control groups. Females accounted for 85.5%. The aOR of having NTS infection were significantly increased in SLE relative to controls (aOR, 9.20; 95% CI, 4.51-18.78) in 1:20 sex-age matching analysis and (aOR, 7.47; 95% CI=2.08-26.82) in propensity score matching analysis. Subgroup analysis indicated that the SLE risk was high among those who dwelled in rural areas; had rheumatoid arthritis, multiple sclerosis, or Sjogren’s syndrome; and developed intensive and severe NTS infection during admission.

**Conclusions:**

Exposure to NTS infection is associated with the development of subsequent SLE in Taiwanese individuals. Severe NTS infection and other autoimmune diseases such as rheumatoid arthritis, multiple sclerosis, or Sjogren’s syndrome also contributed to the risk of developing SLE.

## Highlights

- This population-based, case-control study suggested a significant association between a history of nontyphoidal *Salmonella* (NTS) infection and systemic lupus erythematosus (SLE).
- NTS may be a potential trigger for SLE *via* signaling of proinflammatory cytokines produced by innate and adaptive immune responses.
- A massive data analysis containing approximately 99% of Taiwan’s population of 23 million people

## Introduction

Systemic lupus erythematosus (SLE), whose global prevalence and incidence respectively range from 9–241 and 0.3–23.2 per 1,00,000 person-years (1), is among the top 20 leading causes of death in women aged 5–64 years (2). The multifactorial etiology of SLE, including genetic factors, immune dysfunction-related factors, environmental and hormonal factors, can be deemed as the result of exposome–epigenome–genome interactions (3). Genetic susceptibility alone, however, is insufficient to trigger the onset of SLE, with the evidence of only 24% concordance rates among monozygotic twins with SLE manifestation (4). This indicates that besides genetic factors, the environment also plays a crucial role in the pathophysiology of SLE (5). In addition, heritability, shared (familial) environmental-, and nonshared environmental factors were reported to account for SLE susceptibility in 43.9%, 25.8%, and 30.3% Taiwanese patients, respectively (6).

The major environmental factors influencing SLE development include exposure to ultraviolet radiation, particulate air pollution, trace elements, alcohol use, and infections (7). A correlation between persistent infections and the development of autoimmune diseases has been reported. Infections by viruses, especially Epstein–Barr virus, parvovirus B19, cytomegalovirus, varicella-zoster virus (8), and retrovirus, might play a pivotal pathogenetic role in SLE and are highly associated with SLE (9). Likewise, bacterial infections are also linked to SLE. Gut commensals, for instance, were reported to contribute to the immune pathogenesis of lupus nephritis (10). Additionally, a common anti-DNA idiotype (16/6Id) carried by antibodies against *Mycobacterium tuberculosis* accounts for the antinuclear autoantibody positivity in tuberculosis patients (11), which may lead to autoimmune phenomena (12).

The association between salmonellae infections and the development of autoimmune diseases (13–16) has been reported recently. Salmonella enterica serovar Typhimurium and Enteritidis seem to act more aggressively than other bacterial infections in SLE patients. Other than causing localized gastroenteritis, salmonella infection in SLE patients can cause bacteremia and complications with high mortality rates (17, 18).

Nontyphoidal S*almonellae* (NTS) is a major cause of diarrhea and foodborne gastroenteritis worldwide, and the risk of invasive gastroenteritis is six-fold higher than that of other bacterial infections (19). The global burden of NTS gastroenteritis was estimated to be 94 million cases and 1,55,000 deaths yearly worldwide, with an especially high incidence of 4 cases per 100 persons and over 88,000 associated deaths in 2006 in east Asia (20).

Salmonella infection due to defective Toll-like receptor (TLR)/myeloid differentiation primary response 88 signaling results in disruption of the cellular plasma membrane in B cells, highlighting the importance of correlations between genotype and environmental factors (21).

We hypothesized that a past NTS infection increases the risk of subsequent SLE. As the epidemiological relationship between NTS and SLE has not been clarified, we conducted this original nationwide case-control study to address this important issue.

## Methods

### Data Source

This study was conducted using data from Taiwan’s National Health Insurance Research Database (NHIRD), which consists of original claim data and registration files for almost 99% of Taiwan’s population. Registry for Catastrophic Illness Patients (RFCIP) belongs to a subset of NHIRD that lists diseases requiring long-term care. Patients in RFCIP are exempted from medical expenses as to protect vulnerable beneficiaries. Therefore, the reliability and accuracy of SLE diagnosis received by the enrolled patients were assured. We also employed another subset of NHIRD, the Longitudinal Health Insurance Research Database (LHIRD), in this study. The LHIRD comprises all the original claims data of 1,000,000 people randomly sampled from the year 1997 to 2013 registry of the NHIRD. The positive predictive value of NHIRD claims data can be up to 84.6% with a strict definition of study group selection. In Taiwan, hospital-licensed medical records technicians validated the coding before claiming the reimbursements, and the National Health Insurance Administration authority verified the audit. The Institutional Review Board of Taichung Veterans General Hospital (TCVGH CE14149B-1) approved this study.

### Study Population

**Figure 1:**
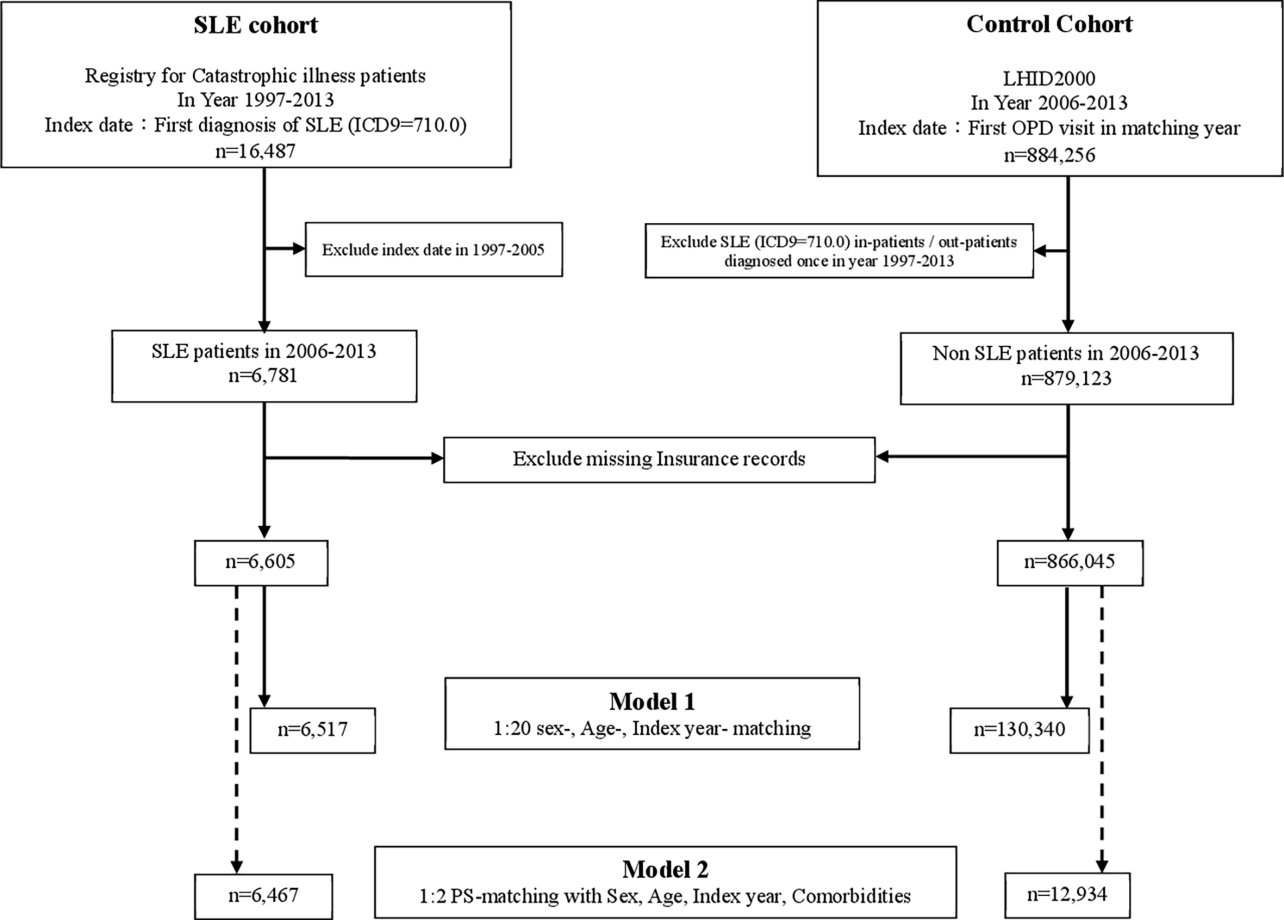
Flowchart. RFCIP, Registry for Catastrophic illness patients; LHID, Longitudinal Health Insurance Research Database; SLE, Systemic lupus erythematosus; OPD, outpatient department; PS-matching, Propensity score matching.

As shown in **Figure 1**, we identified patients who had been diagnosed with SLE by the International Classification of Diseases, Ninth Revision (ICD-9) codes (710.0) in the RFCIP during the period 1997–2013 (n=16,487). The index date was defined as the first date of SLE diagnosis during 2006–2013. Individuals diagnosed with SLE before 2006 were excluded. The control group was randomly selected from LHIRD during 2006–2013, which contained outpatients and inpatients data and was matched at a ratio of 1:20 by age, sex, and index year. Totally, 6,517 patients were included in the SLE group, and 130,340 non-SLE individuals served as the control group. We further performed propensity score matching (PSM) at a 1:2 ratio to minimize the potential confounding effects of sex, age, and selected comorbidities on the incidence of SLE.

### Exposure and Potential Confounders

The exposure of interest in this study was NTS (ICD-9-CM code 003.xx). The exposure/covariate assessment period was set from the year 2000 to the index date. Quantiles were often used in the preliminary assessment of exposure-outcome relationships, and we applied quartiles for cut-offs. To eliminate potential confounding bias, we adjusted for demographic variables and relevant comorbidities whose relative risks are significant to SLE according to previous studies (6, 22). These comorbidities, selected based on at least three outpatient visits or one admission, included psoriasis (ICD-9-CM code 696.1), psoriatic arthropathy (696.0), inflammatory bowel disease (555–556), Crohn’s disease (555), ulcerative colitis (556), ankylosing spondylitis (720.0), Sjogren’s syndrome (710.2), rheumatoid arthritis (714.0), tonsillitis (474.0, 463), psychosocial status (290–319), chronic obstructive pulmonary disease (COPD; 491-492, 493-496), alcohol-related illness (303, 305), chronic liver disease (571.4), cancer (140–239), and multiple sclerosis (340). High-risk drugs (23) for SLE with an administration duration of more than 28 days, such as procainamide (ATC code C01BA02), hydralazine (C02DB02), and isoniazid (J04AC01), were included. Gingival and periodontal diseases (ICD-9-CM code 253) were diagnosed by dentists and included based on the use of one of the following procedures/medications: (1) AHFS code: antibacterials (081200), aminoglycosides (081202), antifungal antibiotics (081204), cephalosporins (081206), miscellaneous beta-lactam antibiotics (081207), chloramphenicol (081208), macrolides (081212), penicillins (081216), tetracyclines (081224), miscellaneous antibiotics (081228), antibiotic composites (081299), antibacterials for eye/ear/nose/throat (520404), and antibacterials for skin/mucous membrane (840404); (2) procedure code: periodontal emergency treatment (91001), subgingival curettage (91006, 91007, 91008), periodontal flap operation (91009, 91010), gingivectomy (91011, 91012, 91013), comprehensive periodontal treatment (91015, 91016); and (3) procedure code: scaling (91003, 91004) over three times a year. We tracked these exposures and comorbidities till the index date of SLE diagnosis.

### Statistical Analysis

We measured the balance of baseline characteristics in the age-matched, sex-matched, and propensity score-matched populations by the absolute standardized difference (ASD), whose value if <0.1 was considered a small difference. Student’s *t*-test was used to compare variables between cases and controls for continuous variables, and chi-square test was used for comparing categorical variables. We used multivariable conditional logistic regression models to estimate the association between NTS and SLE. To further control for potential confounding factors, all models were adjusted for comorbidities and medications that might be associated with SLE. We used the following four models to estimate the effect of NTS on the risk of SLE: (1) model 1, NTS exposure alone, (2) model 2, NTS exposure + demographic variables, (3) model 3, NTS exposure + demographic variables + medical utilization and comorbidities at baseline in age-matched and sex-matched populations, (4) model 4, a conditional logistic model with NTS exposure alone in propensity score-matched population. Furthermore, we performed subgroup analyses by sex, age, urbanization, insurance amount, cumulative cost of NTS-related visits, and number of NTS visits. To improve the reliability of NTS ICD coding, we enrolled only patients using antibiotics at admission for further ascertainment. Conditional logistic regression of adjusted odds ratios (aORs) with 95% confidence intervals (CIs) was conducted. All data analyses were performed using SAS^®^ (version 9.4; SAS Institute, Inc., Cary, NC, USA). Statistical significance level was set at P-value <0.05 in the two-tailed test.

## Results

**Table 1** demonstrates the baseline characteristics of 136,857 and 19,401 patients in 1:20 age–sex matching and in 1:2 PSM analyses, respectively. The mean age of patients in both SLE and non-SLE groups was 37.8 years. There was an obvious female predominance (85.5%) in the SLE population, with the majority aged 20–40 years (43.9%), dwelling in suburban regions (48%), and having tonsillitis (35.4%) and gingival and periodontal diseases (43.9%). The difference in covariates between both groups was well balanced in PSM analysis with the ASD <0.1. The overall ASD revealed a good balance in both SLE and non-SLE groups, whereas it remained mildly uneven in the number of NTS visits subgroup and the interval between the first NTS visit and the index date subgroup with P>0.05 after PSM.

**Table 1:** Baseline characteristics among SLE group and non- SLE group.

	Before PSM (1:20 age–sex matching)				1:2 PSM			
	Non SLE	SLE	P value	ASD	Non SLE	SLE	P value	ASD
	n=130,340	n=6,517			n=12,934	n=6,467		
**Sex**			1.000	0.000			1.000	0.000
Female	111,440 (85.5)	5,572 (85.5)			11,054 (85.5)	5,527 (85.5)		
Male	18,900 (14.5)	945 (14.5)			1,880 (14.5)	940 (14.5)		
**Age**	37.8 ± 17.7	37.8 ± 17.7	1.000		38.5 ± 18.2	37.7 ± 17.7	0.002	
Age group			1.000	0.000			0.078	0.030
<20	20,100 (15.4)	1,005 (15.4)			1,996 (15.4)	1,004 (15.5)		
20≤ age<40	57,180 (43.9)	2,859 (43.9)			5,539 (42.8)	2,846 (44.0)		
40≤Age<60	36,640 (28.1)	1,832 (28.1)			3,616 (28.0)	1,809 (28.0)		
60≤Age	16,420 (12.6)	821 (12.6)			1,783 (13.8)	808 (12.5)		
**Urbanization**			<0.001	0.071			0.901	0.003
Urban	41,683 (32.0)	1,915 (29.4)			3,769 (29.1)	1,902 (29.4)		
Suburban	62,407 (47.9)	3,126 (48)			6,246 (48.3)	3,102 (48.0)		
Rural	26,250 (20.1)	1,476 (22.6)			2,919 (22.6)	1,463 (22.6)		
**Insurance amount (AMT)**			<0.001	0.051			0.874	0.008
AMT=0	43,995 (33.8)	2,246 (34.5)			4,521 (35.0)	2,228 (34.5)		
0<AMT ≤ 18,300	21,120 (16.2)	1,152 (17.7)			2,251 (17.4)	1,145 (17.7)		
18,300<AMT ≤ 28,800	33,848 (26.0)	1,749 (26.8)			3,481 (26.9)	1,736 (26.8)		
28,800<AMT	31,377 (24.1)	1,370 (21.0)			2,681 (20.7)	1,358 (21.0)		
**Co-morbidity^†^**								
Acute anterior uveitis	358 (0.3)	37 (0.6)	<0.001	0.045	59 (0.5)	36 (0.6)	0.344	0.014
PsO	420 (0.3)	55 (0.8)	<0.001	0.069	103 (0.8)	51 (0.8)	0.954	0.001
PsA	31 (0.02)	8 (0.1)	<0.001	0.037	18 (0.1)	6 (0.1)	0.386	0.014
IBD	1,309 (1.0)	89 (1.4)	0.005	0.033	162 (1.3)	88 (1.4)	0.529	0.010
CD	1,203 (0.9)	80 (1.2)	0.013		150 (1.2)	79 (1.2)	0.707	
UC	107 (0.1)	9 (0.1)	0.129		11 (0.1)	9 (0.1)	0.268	
AS	319 (0.2)	53 (0.8)	<0.001	0.078	82 (0.6)	49 (0.8)	0.321	0.015
SS	609 (0.5)	228 (3.5)	<0.001	0.219	379 (2.9)	182 (2.8)	0.650	0.007
RA	897 (0.7)	378 (5.8)	<0.001	0.292	630 (4.9)	331 (5.1)	0.454	0.011
Tonsillitis	43,215 (33.2)	2,310 (35.4)	<0.001	0.048	4,576 (35.4)	2,294 (35.5)	0.899	0.002
Psychosocial status	20,386 (15.6)	1,470 (22.6)	<0.001	0.177	3,050 (23.6)	1,443 (22.3)	0.048	0.030
COPD	15,625 (12.0)	1,029 (15.8)	<0.001	0.110	2,091 (16.2)	1,011 (15.6)	0.339	0.015
Alcohol related illness	603 (0.5)	50 (0.8)	<0.001	0.039	78 (0.6)	50 (0.8)	0.168	0.021
Chronic liver disease	7,521 (5.8)	600 (9.2)	<0.001	0.131	1,207 (9.3)	590 (9.1)	0.636	0.007
Cancer	22,388 (17.2)	1,484 (22.8)	<0.001	0.140	3,028 (23.4)	1,466 (22.7)	0.248	0.018
Multiple sclerosis	19 (0.01)	7 (0.1)	<0.001	0.038	11 (0.1)	5 (0.1)	0.860	0.003
Gingival and periodontal diseases	49,488 (38.0)	2,861 (43.9)	<0.001	0.121	5,749 (44.4)	2,840 (43.9)	0.481	0.011
**SLE risk drugs† use ≥ 28 days**	2,440 (1.9)	171 (2.6)	<0.001		290 (2.2)	169 (2.6)	0.109	
**NTS (Outpatients or Inpatients)**	1,164 (0.9)	78 (1.2)	0.012	0.030	135 (1.0)	78 (1.2)	0.306	0.015
**Number of NTS visits (Outpatients or Inpatients)**			0.027				0.475	
Non NTS	129,176 (99.1)	6,439 (98.8)			12,799 (99.0)	6,389 (98.8)		
1 visit	724 (0.6)	45 (0.7)			84 (0.6)	45 (0.7)		
>1 visit	440 (0.3)	33 (0.5)			51 (0.4)	33 (0.5)		
**Cumulative cost of NTS-related visits (New Taiwan dollars)**			<0.001				0.026	
non NTS	129,176 (99.1)	6,439 (98.8)			12,799 (99.0)	6,389 (98.8)		
dollars ≤ 333	298 (0.2)	14 (0.2)			36 (0.3)	14 (0.2)		
333<dollars ≤ 425	322 (0.2)	13 (0.2)			38 (0.3)	13 (0.2)		
425<dollars ≤ 1064	267 (0.2)	18 (0.3)			28 (0.2)	18 (0.3)		
1064<dollars	277 (0.2)	33 (0.5)			33 (0.3)	33 (0.5)		
**Interval between the first NTS visit and the index date**			0.016				0.186	
non NTS	129,176 (99.1)	6,439 (98.8)			12,799 (99.0)	6,389 (98.8)		
years ≤ 3.53	289 (0.2)	22 (0.3)			31 (0.2)	22 (0.3)		
3.53<years ≤ 6.08	286 (0.2)	24 (0.4)			32 (0.2)	24 (0.4)		
6.08<years ≤ 8.62	298 (0.2)	12 (0.2)			38 (0.3)	12 (0.2)		
8.62<years	291 (0.2)	20 (0.3)			34 (0.3)	20 (0.3)		
**Number of NTS visits, 3 months interval (Outpatients or Inpatients)**			0.022				0.589	
Non NTS	129,176 (99.1)	6,439 (98.8)			12,799 (99)	6,389 (98.8)		
1 visit	835 (0.6)	60 (0.9)			103 (0.8)	60 (0.9)		
>1 visit	329 (0.3)	18 (0.3)			32 (0.2)	18 (0.3)		
**Number of NTS visits, 3 months interval (Inpatients only)**								
Non NTS	130,255 (99.9)	6,497 (99.7)	<0.001		12,919 (99.9)	6,447 (99.7)	0.003	
1 visit	84 (0.1)	20 (0.3)			15 (0.1)	20 (0.3)		
>1 visit	1 (0.001)	0 (0.0)			0 (0.0)	0 (0.0)		
≥1 visit	85 (0.1)	20 (0.3)			15 (0.1)	20 (0.3)		
Non NTS	130,255 ( )	6,497 ( )			12,919 ( )	6,447 ( )		
≥1 visit	85 [ ]	20 [ ]			15 [ ]	20 [ ]		
No antibiotics use during visit	25 (0.02) [29.4]	3 (0.05) [15.0]	(<0.001) [0.147]		6 (0.05) [40.0]	3 (0.05) [15.0]	(0.006) [0.175]	
IV antibiotics use during visit	30 (0.02) [35.3]	5 (0.1) [25.0]			5 (0.04) [33.3]	5 (0.1) [25.0]		
Oral antibiotics use during visit	6 (0.005) [7.1]	1 (0.02) [5.0]			1 (0.01) [6.7]	1 (0.02) [5.0]		
IV + Oral antibiotics use during visit	24 (0.02) [28.2]	11 (0.2) [55.0]			3 (0.02) [20.2]	11 (0.2) [55.0]		
No antibiotics use ≥ 3 days during visit	26 (0.02) [30.6]	4 (0.1) [20.0]	(<0.001) [0.151]		6 (0.05) [40.0]	4 (0.1) [20.0]	(0.008) [0.216]	
IV antibiotics use ≥ 3 days during visit	30 (0.02) [35.3]	4 (0.1) [20.0]			5 (0.04) [33.3]	4 (0.1) [20.0]		
Oral antibiotics use ≥ 3 days during visit	5 (0.004) [5.9]	1 (0.02) [5.0]			1 (0.01) [6.7]	1 (0.02) [5.0]		
IV + Oral antibiotics use ≥ 3 days during visit	24 (0.02) [28.2]	11 (0.2) [55.0]			3 (0.02) [20.0]	11 (0.2) [55.0]		
**Number of NTS visits, 3 months interval (Inpatients only with antibiotics use)** ^†^			<0.001				<0.001	
Non NTS	130,280 (99.95)	6,500 (99.7)			12,925 (99.9)	6,450 (99.7)		
1 visit	59 (0.05)	17 (0.3)			9 (0.1)	17 (0.3)		
>1 visit	1 (0.001)	0 (0.0)			0 (0.0)	0 (0.0)		
**Number of NTS visits, 3 months interval (Inpatients only with intravenous antibiotics use)** ^†^			<0.001				<0.001	
Non NTS	130,286 (100)	6,501 (99.8)			12,926 (99.9)	6,451 (99.8)		
1 visit	53 (0.04)	16 (0.2)			8 (0.1)	16 (0.2)		
>1 visit	1 (0.001)	0 (0.0)			0 (0.0)	0 (0.0)		
**Number of NTS visits, 3 months interval (Inpatients only with oral antibiotics use)** ^†^			<0.001				<0.001	
Non NTS	130,310 (100)	6,505 (99.8)			12,930 (100)	6,455 (99.8)		
1 visit	30 (0.02)	12 (0.2)			4 (0.03)	12 (0.2)		
>1 visit	1 (0.001)	0 (0.0)			0 (0.0)	0 (0.0)		
**Number of NTS visits, 3 months interval (Inpatients only with antibiotics use ≥ 3 days)** ^†^			<0.001				0.001	
Non NTS	130,281 (100)	6,501 (99.8)			12,925 (99.9)	6,451 (99.8)		
1 visit	58 (0.04)	16 (0.2)			9 (0.1)	16 (0.2)		
>1 visit	1 (0.001)	0 (0.0)			0 (0.0)	0 (0.0)		
**Number of NTS visits, 3 months interval (Inpatients only with intravenous antibiotics use ≥ 3 days)** ^†^			<0.001				0.001	
Non NTS	130,286 (100)	6,502 (99.8)			12,926 (99.9)	6,452 (99.8)		
1 visit	53 (0.04)	15 (0.2)			8 (0.1)	15 (0.2)		
>1 visit	1 (0.001)	0 (0.0)			0 (0.0)	0 (0.0)		
**Number of NTS visits, 3 months interval (Inpatients only with oral antibiotics use ≥ 3 days)** ^†^			<0.001				<0.001	
Non NTS	130,311 (100)	6,505 (99.8)			12,930 (100)	6,455 (99.8)		
1 visit	29 (0.02)	12 (0.2)			4 (0.03)	12 (0.2)		
>1 visit	0 (0.0)	0 (0.0)			0 (0.0)	0 (0.0)		

^†^Comorbidity and SLE risk drugs (Procainamide, Hydralazine, Isoniazid) were identified from year 2000 to the index date.

PSM, Propensity score matching; ASD, Absolute standardized difference; SLE, Systemic lupus erythematosus; PsO, Psoriasis; PsA, Psoriatic arthropathy; IBD, Inflammatory bowel disease; CD, Crohn’s disease; UC, Ulcerative colitis; AS, Ankylosing spondylitis; SS, Sjogren’s syndrome; RA, Rheumatoid arthritis; COPD, Chronic obstructive pulmonary disease.

⍰ Antibiotics: 1. Quinolone or 2. Cefixime/Ceftriaxone/Cefotaxime, or 3. Trimethoprime/Sulfamethoxazole, or 4. Ampicillin/Augmentin (Ampicillin +/Clavulanic acid)/Unasyn (Ampicillin + Salbactam).

PSM, Propensity score matching; ASD, Absolute standardized difference; SLE, Systemic lupus erythematosus; NTS, Nontyphoidal Salmonella.

**Table 2** shows aORs and 95% CIs estimated by conditional logistic regression models, with 1:20 age–sex matching and PSM analyses in NTS patients enrolled through ambulatory care or admission. The aOR for SLE was 1.35 (95% CI=1.07-1.70) in model 1 and 1.35 (95% CI=1.07-1.71) in model 2. Dwelling in rural (aOR=1.20; 95% CI=1.12-1.29) and suburban (aOR=1.08; 95% CI=1.02-1.14) areas was associated with a higher risk of SLE in NTS patients than dwelling in urban areas (model 2). NTS patients with rheumatoid arthritis (aOR=7.11; 95% CI=6.22-8.12), multiple sclerosis (aOR=5.44; 95% CI=2.15-13.77), and Sjogren’s syndrome (aOR=5.03; 95% CI=4.24-5.97) had a higher risk of SLE in model 3. The OR in PSM analysis revealed no significant difference between the NTS and control groups in terms of the future incidence of SLE (aOR=1.46; 95% CI=0.93-2.28) in patients who were enrolled through ambulatory care or admission (model 4).

**Table 2:** Unadjusted and adjusted odds ratios for SLE and exposure to NTS.

		1:20 age-matched and sex-matched population			1:2 PSM population
	Univariable	Model 1: NTS exposure alone	Model 2: NTS exposure + demographic variables	Model 3: model 2 + medical utilization and comorbidities at baseline	Model 4: conditional logistic model with NTS exposure alone
**NTS (Outpatients or Inpatients)**	1.35 (1.07–1.70)	1.35 (1.07–1.70)	1.35 (1.07–1.71)	1.25 (0.99–1.59)	1.46 (0.93–2.28)
**Urbanization**					
Urban	Ref.		Ref.	Ref.	
Suburban	1.09 (1.03–1.16)		1.08 (1.02–1.14)	1.07 (1.01–1.14)	
Rural	1.23 (1.15–1.32)		1.20 (1.12–1.29)	1.18 (1.10–1.27)	
**Insurance amount (AMT)**					
AMT=0	Ref.		Ref.	Ref.	
0<AMT ≤ 18,300	1.04 (0.95–1.13)		1.03 (0.95–1.12)	1.03 (0.94–1.12)	
18,300<AMT ≤ 28,800	0.97 (0.90–1.05)		0.96 (0.89–1.04)	0.95 (0.88–1.03)	
28,800<AMT	0.81 (0.75–0.88)		0.82 (0.76–0.90)	0.82 (0.75–0.89)	
**Co-morbidity^†^**					
Acute anterior uveitis	2.08 (1.48–2.92)			1.73 (1.21–2.47)	
PsO	2.64 (1.99–3.51)			2.27 (1.67–3.09)	
PsA	5.21 (2.39–11.39)			1.75 (0.71–4.32)	
IBD	1.37 (1.10–1.70)			1.22 (0.98–1.52)	
AS	3.35 (2.50–4.49)			1.83 (1.32–2.54)	
SS	8.01 (6.84–9.37)			5.03 (4.24–5.97)	
RA	9.52 (8.38–10.80)			7.11 (6.22–8.12)	
Tonsillitis	1.12 (1.06–1.18)			1.02 (0.97–1.08)	
Psychosocial status	1.65 (1.55–1.76)			1.37 (1.28–1.47)	
COPD	1.42 (1.32–1.52)			1.20 (1.11–1.29)	
Alcohol related illness	1.67 (1.25–2.24)			1.16 (0.86–1.57)	
Chronic liver disease	1.71 (1.56–1.87)			1.41 (1.28–1.55)	
Cancer	1.48 (1.39–1.57)			1.33 (1.25–1.42)	
Multiple sclerosis	7.37 (3.10–17.53)			5.44 (2.15–13.77)	
Gingival and periodontal diseases	1.30 (1.23–1.37)			1.24 (1.18–1.31)	
**SLE risk drugs use ≥ 28 days**	1.45 (1.23–1.71)			1.30 (1.09–1.53)	

^†^Comorbidity and SLE risk drugs (Procainamide, Hydralazine, Isoniazid) were identified from year 2000 to the index date.

PSM, Propensity score matching; SLE, Systemic lupus erythematosus; NTS, Nontyphoidal Salmonella; PsO, Psoriasis; PsA, Psoriatic arthropathy; IBD, Inflammatory bowel disease; CD, Crohn’s disease; UC, Ulcerative colitis; AS, Ankylosing spondylitis; SS, Sjogren’s syndrome; RA, Rheumatoid arthritis; COPD, Chronic obstructive pulmonary disease.

**Table 3** demonstrates the subgroup analysis of conditional logistic regression models.

**Table 3:** Unadjusted and adjusted odds ratios for SLE and exposure to NTS.

		1:20 age-matched and sex-matched population			1:2 PSM population
	Univariable	Model 1: NTS exposure alone	Model 2: NTS exposure + demographic variables	Model 3: model 2 + medical utilization and comorbidities at baseline	Model 4: conditional logistic model with NTS exposure alone
**Number of NTS visits (Outpatients or Inpatients)**					
non NTS	Ref.	Ref.	Ref.	Ref.	Ref.
1 visit	1.25 (0.92–1.69)	1.25 (0.92–1.69)	1.26 (0.93–1.70)	1.15 (0.84–1.56)	1.35 (0.81–2.24)
>1 visit	1.99 (1.24–3.19)	1.51 (1.06–2.15)	1.52 (1.06–2.16)	1.43 (1.00–2.05)	1.63 (0.93–2.86)
**Cumulative cost of NTS-related visits (New Taiwan dollars) (Outpatients or Inpatients)**					
non NTS	Ref.	Ref.	Ref.	Ref.	Ref.
dollars ≤ 333	0.94 (0.55–1.61)	0.94 (0.55–1.61)	0.95 (0.56–1.63)	0.86 (0.50–1.50)	0.89 (0.43–1.83)
333<dollars ≤ 425	0.81 (0.47–1.41)	0.81 (0.47–1.41)	0.81 (0.47–1.42)	0.74 (0.42–1.30)	0.81 (0.38–1.71)
425<dollars ≤ 1064	1.36 (0.84–2.19)	1.36 (0.84–2.19)	1.37 (0.85–2.21)	1.36 (0.84–2.20)	1.59 (0.79–3.23)
1064<dollars	2.40 (1.67–3.45)	2.40 (1.67–3.45)	2.40 (1.67–3.45)	2.15 (1.48–3.11)	2.29 (1.30–4.03)
**Interval between the first NTS visit and the index date (Outpatients or Inpatients)**					
non NTS	Ref.	Ref.	Ref.	Ref.	Ref.
years ≤ 3.53	1.53 (0.99–2.36)	1.53 (0.99–2.36)	1.52 (0.99–2.35)	1.46 (0.94–2.26)	1.64 (0.85–3.17)
3.53<years ≤ 6.08	1.68 (1.11–2.56)	1.68 (1.11–2.56)	1.69 (1.11–2.57)	1.51 (0.99–2.32)	1.79 (0.94–3.39)
6.08<years ≤ 8.62	0.81 (0.45–1.44)	0.81 (0.45–1.44)	0.82 (0.46–1.46)	0.75 (0.42–1.36)	0.75 (0.34–1.68)
8.62<years	1.38 (0.88–2.18)	1.38 (0.88–2.18)	1.40 (0.88–2.20)	1.30 (0.82–2.06)	1.44 (0.72–2.88)
**Number of NTS visits (3 months interval, Outpatients or Inpatients)**					
non NTS	Ref.	Ref.	Ref.	Ref.	Ref.
Visits=1	1.44 (1.11–1.88)	1.44 (1.11–1.88)	1.45 (1.11–1.89)	1.35 (1.03–1.77)	1.48 (0.91–2.40)
Visits≥2	1.10 (0.68–1.77)	1.10 (0.68–1.77)	1.11 (0.69–1.79)	1.02 (0.63–1.65)	1.39 (0.70–2.78)
**Number of NTS visits ≥ 1 (3 months interval, Inpatients only)**					
	4.77 (2.92–7.78)	4.77 (2.92–7.78)	4.77 (2.92–7.79)	4.04 (2.44–6.68)	2.88 (1.43–5.82)
Non NTS	Ref	Ref	Ref	Ref	Ref
No antibiotics use during visit ^†^	2.43 (0.73–8.07)	2.43 (0.73–8.07)	2.44 (0.78–8.12)	1.84 (0.53–6.32)	1.18 (0.29–4.83)
IV antibiotics use during visit ^†^	3.38 (1.31–8.73)	3.38 (1.31–8.73)	3.33 (1.29–8.59)	2.89 (1.10–7.61)	2.02 (0.58–6.99)
Oral antibiotics use during visit ^†^	3.34 (0.40–27.77)	3.34 (0.40–27.77)	3.34 (0.40–27.77)	2.80 (0.33–23.70)	2.00 (0.13–31.98)
IV + Oral antibiotics use during visit ^†^	9.21 (4.51–18.79)	9.21 (4.51–18.79)	9.36 (4.58–19.11)	8.36 (4.01–17.43)	7.38 (2.06–26.49)
No antibiotics use ≥ 3 days during visit ^†^	3.13 (1.09–9.00)	3.13 (1.09–9.00)	3.13 (1.09–9.02)	2.45 (0.82–7.30)	1.54 (0.42–5.58)
IV antibiotics use ≥ 3 days during visit ^†^	2.70 (0.95–7.68)	2.70 (0.95–7.68)	2.66 (0.94–7.57)	2.28 (0.79–6.61)	1.64 (0.44–6.14)
Oral antibiotics use ≥ 3 days during visit ^†^	4.00 (0.47–34.24)	4.00 (0.47–34.24)	3.99 (0.47–34.16)	3.19 (0.36–27.91)	2.00 (0.13–31.98)
IV + Oral antibiotics use ≥ 3 days during visit ^†^*	9.20 (4.51–18.78)	9.20 (4.51–18.78)	9.35 (4.58–19.10)	8.35 (4.00–17.42)	7.47 (2.08–26.82)
**Number of NTS visits ≥ 1 (3 months interval, Inpatients only with antibiotics use)** ^†^					
	5.71 (3.33–9.80)	5.71 (3.33–9.80)	5.71 (3.32–9.80)	5.00 (2.88–8.70)	3.78 (1.68–8.48)
**Number of NTS visits ≥ 1 (3 months interval, Inpatients only with antibiotics use ≥ 3 days)** ^†^					
	5.46 (3.14–9.50)	5.46 (3.14–9.50)	5.46 (3.14–9.50)	4.74 (2.69–8.36)	3.56 (1.57–8.05)

*Main model.

^†^Antibiotics: 1. Quinolone or 2. Cefixime/Ceftriaxone/Cefotaxime, or 3. Trimethoprime/Sulfamethoxazole, or 4. Ampicillin/Augmentin (Ampicillin +/Clavulanic acid)/Unasyn (Ampicillin + Salbactam).

PSM, Propensity score matching; SLE, Systemic lupus erythematosus; NTS, Nontyphoidal Salmonella.

### NTS Patients Enrolled Through Ambulatory Care or Admission

Patients who visited the hospital for NTS more than one time either through ambulatory care or admission had a higher risk of future SLE, with an aOR of 1.51 (95% CI=1.06-2.15) in model 1, 1.52 (95% CI=1.06-2.16) in model 2, and 1.43 (95% CI=1.00-2.05) in model 3. Furthermore, NTS patients with intensive visits within 3 months were more predisposed to SLE development than patients who had long-term visits, with aOR of 1.44 (95% CI=1.11-1.88) in model 1, 1.45 (95% CI=1.11-1.89) in model 2, and 1.35 (95% CI=1.03-1.77) in model 3.

A higher cumulative cost of NTS-related visits (>1064 New Taiwan Dollars) was associated with a higher SLE risk, with an aOR of 2.40 (95% CI=1.67-3.45) in model 1, 2.40 (95% CI=1.67-3.45) in model 2, 2.15 (95% CI=1.48-3.11) in model 3, and 2.29 (95% CI=1.30-4.03) in model 4.

Analysis of the interval between the first NTS visit and the index date revealed that an interval of 3.53–6.08 years was associated with a higher SLE risk, with an aOR of 1.68 (95% CI=1.11-2.56) in model 1 and 1.69 (95% CI=1.11-2.57) in model 2.

### NTS Patients Enrolled Through Admission Only

Patients who visited the hospital for NTS at least one time through admission had a higher risk of future SLE, with an aOR of 4.77 (95% CI=2.92-7.78) in model 1, 4.77 (95% CI=2.92-7.79) in model 2, 4.04 (95% CI=2.44-6.68) in model 3, and 2.88 (95% CI=1.43-5.82) in model 4. In the subgroups of antibiotic use, Intravenous (IV) antibiotics use during visit showed significant risk in model 1 (aOR=3.38, 95% CI= 1.31-8.73), model 2 (aOR=3.33, 95% CI= 1.29-8.59), and model 3 (aOR=2.89, 95% CI= 1.10-7.61). In the subgroups of “IV + oral antibiotics use (no matter ≥ 3 days or not) during visit” showed significant higher risk in 4 models, with aOR ranged from 7.38 to 9.36. Especially in “IV + oral antibiotics use ≥ 3 days during visit” subgroup (main model), aOR of 9.20 (95% CI=4.51-18.78), and 7.47 (95% CI= 2.08-26.82) were observed using 1:20 sex-age matching and 1:2 PSM, respectively.

In addition, NTS patients who were admitted with antibiotics use had a higher risk of SLE, with an aOR of 5.71 (95% CI=3.33-9.80) in model 1; 5.71 (95% CI=3.32-9.80) in model 2; 5.00 (95% CI=2.88-8.70) in model 3; 3.78 (95% CI=1.68-8.48) in model 4.

Finally, NTS patients who were admitted with antibiotics use for at least 3 days had a higher risk of SLE, with an aOR of 5.46 (95% CI=3.14-9.50) in model 1, 5.46 (95% CI=3.14-9.50) in model 2, 4.74 (95% CI=2.69-8.36) in model 3, and 3.56 (95% CI=1.57-8.05) in model 4.

## Discussion

In this nationwide, population-based case-control study, we found that the risk of new-onset SLE was 9.20-fold and 7.47-fold higher in patients with a history of NTS than in matched controls by 1:20 sex–age matching and PSM analyses, respectively. Consistent with epidemiological observations, Comorbidities such as Sjogren’s syndrome (24) (ASD = 0.219), Rheumatoid arthritis (25) (ASD = 0.292), Psychosocial status (26) (ASD = 0.177), gingival and periodontal disease (27) (ASD = 0.121) etc. were significantly more predominant in SLE patients than in general population (**Table 1**).

Despite different models rendered different odds ratio in **Table 2**, our data implied that SLE was prone to rise among NTS-infected patients who had Sjogren’s syndrome, Rheumatoid arthritis, Multiple sclerosis, and who lived in rural area rather than suburban and urban area. In **Table 3**, among ambulatory care or admission patients, the more intensive NTS visit in one NTS infection episode, the higher of NTS cumulative cost gave rise to higher odds ratio of future SLE, which largely occurred among 3 to 6 years after NTS infection. Among admission only patients, the more critical NTS infection is (who needed hospitalization and with both IV and oral antibiotics use), the higher odds of future SLE will be. Despite study illustrated that Salmonellosis of long duration was characterized by hyperactivity of autoimmune reactions (28), there was no advanced description on the duration from severe Salmonellosis to the onset of autoimmune disease.

Environmental exposures other than infections might also depend on the level of urbanization. First, the smoking behavior of Taiwanese individuals was estimated to be 71.63% and was largely distributed (47.94%) in suburban area (29), which correlated with our observation. Second, dwelling in areas in which the content of particulate matter (2.5 µm) was over 35 µg/m³ was associated with 15% higher long-term mortality risk according to the air quality guideline (30); such areas included many suburban and rural areas in Taiwan in 2013 (31) and are linked to many autoimmune diseases (7). Third, heavy metals were largely spatially distributed in the soil of rural townships during 1982-1986 in Taiwan (32). Major pollution events (33) in the past, such as aluminum slag, electric arc furnace dust pollution, and rice cadmium toxicity, largely in rural areas possibly trigger the onset of SLE or aggravate the condition. Last, low-income households that can afford only low insurance amounts are mainly located in rural areas (34), and they accounted for the higher prevalence of SLE in this study.

Other autoimmune diseases, such as Sjogren’s syndrome, Rheumatoid arthritis, Multiple sclerosis, were prone to develop high risk of SLE in NTS infected patients in our study. Changes in symbiosis and dysbiosis of microbiome could flare autoimmune diseases (35). In patients with Sjogren’s syndrome, the changes of oral microbiome in glandular and mucosal tissues brought about activated plasma cell and CD4+ cells that ruined the secretory acini of salivary glands. Similarly, increased levels of IFN-γ and IL-17 caused by infiltration of CD4+ cells and dendritic cells in the conjunctiva causing ocular disease (36). On the other hand, in patients with Rheumatoid arthritis, dental, saliva, or gut microbiome was distinguished from healthy controls (37). Its HLA-DRB1 molecules, trans-membrane glycoproteins of macrophages and dendritic cells that present antigens to T cells, appear to forge the microbiome signature and build an inflammatory environment in animal study (38). Furthermore, gut microbiome shown to be altered in patients with relapsing Multiple sclerosis, causing changes of the metabolic pathways or the Gut-associated lymphoid tissues (GALTs) and aggravation of inflammatory demyelination in central nervous system (39).

Among environmental factors in SLE pathogenesis, microbiome has gained much attention in recent years. The mechanism by which NTS infection increases the risk of SLE remains unclear and disputed. Yet, we propose that long-term NTS stimulates abundant proinflammatory cytokines and high oxidative stress through innate and adaptive immune response, leading to the onset of SLE.

### Interaction Between Innate and Adaptive Immune Systems

After reaching the lower intestine, salmonella bacteria translocate across Peyer’s patches where they are engulfed by phagocytic cells. Salmonella are recognized by pattern-recognition receptors, triggering cascades toward the induction of interleukin (IL)-1β, IL-18, and IL-23; cell pyroptosis and inefficient clearance of phagocytized bacteria (40). Infections by bacteria such as *Escherichia coli* and *Salmonella* are well-known triggers of pyroptosis (41), which may play a pathogenic role in releasing host nuclear autoantigens in SLE (18).

#### Type I Interferon (IFN-I)

The autoantigens generated from uncleared apoptotic cells and neutrophil extracellular traps may induce the proliferation and differentiation of autoreactive B cells to produce autoantibodies, which then participate in the formation of immune complexes and tissue injuries. Both autoantigens and immune complexes promote the production of IFN-I by activating plasmacytoid dendritic cell signaling *via* amyloid/DNA complexes (42, 43). IFN-I hampers the clearance of apoptotic cells by macrophages and induces B cells to produce autoantibodies (44). In addition, the immune complexes containing antinuclear autoantibodies could provoke plasmacytoid dendritic cells to produce more IFN-I, leading to SLE (45, 46). Therefore, IFN-I has been recognized as the central pathogenic cytokine in SLE onset (47).

#### IL-17, IL-18, and IFN-γ

During NTS infection, there is a massive release of proinflammatory cytokines including IL-23 and IL-18 (40). Antigen-presenting cells produce IL-23, which induces and maintains the activation of Th17 cells (48), and induces various innate and adaptive immune response cells, including innate lymphoid cells (49) and Th17 cells (50) to release the proinflammatory cytokines IL-17 and IL-22. IL-17, in conjunction with B cell activating factor, promotes the activation and proliferation of B cells along with their antibody production and class switching (51, 52). Lupus patients have been reported to have increased serum levels of IL-17 (53) and IL-17-producing cells in the peripheral blood (54, 55). IL-18 is vital in stimulating Th1 cells to release IFN-γ (56), which plays a critical role in macrophage activation for the control of persistent salmonella infection (57–59). At the same time, the elevation of IL-17 and IL-18 in SLE patients may trigger the inflammatory process, and an increase in the IL-18/IL-4 ratio and mRNA levels of IFN-γ/IL-4 (60) tilts the cytokine profile toward inflammatory cascades (53).

In real world practice, we prescribed intravenous antibiotics to patients with severe NTS infection and transited to oral antibiotics when discharged in Taiwan (61, 62). The optimal antibiotic treatment duration for severe NTS gastrointestinal infection was 3-7 days and for NTS bacteremia ranges from 2 weeks (in healthy individuals) to 6 weeks (in immunocompromised patients) (63). Therefore, the data in “IV + Oral antibiotics use ≥ 3 days during visit” group (main model) was more persuasive than that in other groups. Despite low sample size gave rise to low statistical power, the high aOR (aOR=7.47, 95% CI= 2.08-26.82) in “IV + Oral antibiotics use ≥ 3 days during visit” subgroup using PSM strongly implied a correlation between NTS infection and future SLE development.

The advantages of using NHIRD in research include the availability of a large sample size, population-based data, and long-term comprehensive follow-up data. Our study is representative of the general population, and the possible measurable confounding factors of SLE were adjusted through matching with age, sex, demographic variables, and comorbidities through PSM. In addition, we strengthen the validity of the coding of NTS-associated diseases by filtering the NTS patients into those for whom only admission records data were available and those who were administered antibiotics to treat NTS infection. Therefore, the diagnosis of symptomatic NTS infection in our study population can be considered to be reliable.

Several limitations should be considered when interpreting our findings. First, epidemiologic evaluation of evident NTS infection was challenging as many infections were not clinically recognized. The use of retrospective ICD-9-based methods to select study groups of NTS infection might have led to selection bias. Second, NHIRD lacked information on smoking status, a well-known confounder of SLE and the most common risk factor for COPD development (64). Therefore, we managed to apply COPD prevalence as a proxy for smoking status (29). Third, non-differential misclassification bias on the diagnosis of NTS, a dichotomous variable, may exist. However, such bias always drives the result toward the null, and attenuates real effect estimates (65). Fourth, this was a single-country evaluation. Thus, our findings may not be applicable to non-Asian ethnic groups. Finally, the relation between symptomatic NTS and SLE was only demonstrated based on epidemiological evidence.

## Conclusion

Exposure to NTS infection may be associated with an increased risk SLE and the risk increased with the individual severity of NTS infection. More epidemiologic and *in vivo* investigations are warranted to examine whether causality present between NTS infection and its trigger on SLE.

## Data Availability Statement

The original contributions presented in the study are included in the article/supplementary material. Further inquiries can be directed to the corresponding authors.

## Ethics Statement

The Institutional Review Board of Taichung Veterans General Hospital (TCVGH CE14149B-1) approved this study. Written informed consent for participation was not required for this study in accordance with the national legislation and the institutional requirements.

## Author Contributions

Study conception and design: Y-MH, RC, H-HC, JC-CW. Acquisition of data: H-HC. Analysis and interpretation of data: T-YT, C-YY, Y-MH, RC, H-HC, JC-CW. Writing (original draft preparation): T-YY, C-YY. Writing (review and editing): T-YT, C-YY, H-HC, Y-MH, RC, JC-CW. All authors contributed to the article and approved the submitted version.

## Conflict of Interest

The authors declare that the research was conducted in the absence of any commercial or financial relationships that could be construed as a potential conflict of interest.

## Publisher’s Note

All claims expressed in this article are solely those of the authors and do not necessarily represent those of their affiliated organizations, or those of the publisher, the editors and the reviewers. Any product that may be evaluated in this article, or claim that may be made by its manufacturer, is not guaranteed or endorsed by the publisher.
